# Naturally occurring mutations to HCV protease inhibitors in treatment-naïve patients

**DOI:** 10.1186/1743-422X-9-245

**Published:** 2012-10-24

**Authors:** Stefania Paolucci, Loretta Fiorina, Antonio Piralla, Roberto Gulminetti, Stefano Novati, Giorgio Barbarini, Paolo Sacchi, Marta Gatti, Luca Dossena, Fausto Baldanti

**Affiliations:** 1 Molecular Virology Unit, Virology and Microbiology Department, Fondazione IRCCS Policlinico San Matteo, Pavia, 27100, Italy; 2 Institute of Infectious Diseases, University of Pavia, Pavia, 27100, Italy; 3 Division of Infectious and Tropical Diseases, Fondazione IRCCS Policlinico San Matteo, Pavia, 27100, Italy

**Keywords:** Hepatitis C virus, HCV baseline resistance, Protease inhibitors, HIV/HCV co-infection, Genetic diversity

## Abstract

**Background:**

Protease inhibitors (PIs) to treat hepatitis C (HCV) virus infection have been approved and others are under development.

**Results:**

The aims of this study were to illustrate natural polymorphisms in the HCV protease and measure the frequency of PI resistance mutations in different HCV genotypes from PI-naïve patients.

Direct sequencing of HCV NS3/4A protease was performed in 156 HCV patients naïve to PIs who were infected with genotype 1a (n = 31), 1b (n = 39), 2 (n = 30), 3 (n = 33) and 4 (n = 23).

Amino acid (aa) substitutions associated with HCV PI resistance were found in 17/156 (10.8%) sequences. Mutations V36L, T54S, V55A/I, and Q80K/L were observed in 29% of patients with genotype 1a, and V55F, Q80L/N and M175L in 10% of patients with genotype 1b. The mutation V158M was found in 3% of patients with genotype 2, D168Q was present in 100% of patients with genotype 3 and D168E was observed in 13% of patients with genotype 4. In addition, multiple aa polymorphisms not associated with PI resistance were detected in patients with genotypes 1a, 1b and 4.

**Conclusions:**

Although major PI resistance mutations were not detected, other resistance mutations conferring low level resistance to PIs together with a number of natural polymorphisms were observed in proteases of PI naïve HCV patients. A more extensive analysis is needed to better evaluate the impact of baseline resistance and compensatory mutations in the efficacy of HCV PI treatment.

## Background

Hepatitis C virus (HCV) infects more than 170 million people worldwide [1]. Treatment with pegylated interferon-α and ribavirin is burdened by adverse reactions in a significant proportion of patients [2] and a sustained virological response is achieved in only 50% of patients infected with genotype 1 [3] and 80% of patients infected with genotype 2 [4]. Recently, inhibitors of HCV non-structured serine –protease 3 (NS3/4A) have been approved (Telaprevir, Boceprevir), and others are under development (TMC435350, ITMN191, SCH900518, MK7009, BI-201335, MK5172, GS-9256, ABT 45, BMS-791325 and ACH-1625) [5-9]. However, selection of drug-resistant HCV variants has already been reported in protease inhibitor (PI)-treated individuals [7-14]. The degree of resistance appears to be related both to mutations at specific NS3 positions and to changes in amino acid (aa) residues [5,7,15].

The high HCV replication rate and lack of a proof-reading mechanism determine a natural variability, which promotes the rapid emergence of drug-resistant variants [15]. Natural aa changes in NS3 associated with reduced drug susceptibility have been observed in treatment naïve patients [10,16,17]. However, the clinical impact of baseline resistance and its influence on the ability of the virus to replicate *in vivo* remain unclear [15,16]. Recently, the sporadic transmission of naturally occurring NS3 resistance mutations was reported [16]. In addition, the impact of the frequency of baseline HCV PI resistance mutations in HIV/HCV co-infected patients with respect to HCV mono-infected patients is still debated [17-19]. The frequency of naturally occurring NS3 aa substitutions associated with PI resistance in treatment naïve HCV patients infected with genotypes 1, 2, 3 and 4 was investigated.

## Materials and methods

HCV PI-naive patients referred to our hospital between 2010 and 2011 were included in the study. Patients were stratified according to HCV genotype and a comparable number of patients infected with HCV genotypes 1a, 1b, 2, 3 and 4 were sequentially enrolled in the study. Most (75%) were treated with pegylated Interferon-α and Ribavirin, while none had ever been treated with a PI for hepatitis C. For NS3 sequencing, surplus serum samples were prospectively collected from each patient. HCV genotypes were defined using the Versant HCV Genotype 2.0 Assay LiPA (Siemens Healthcare Diagnostic Inc., Tarrytown, NY USA). The NS3 region was sequenced to further subtype HCV strains and identify genotypes 1a/1b. Data were analyzed with the Blast program (http://blast.ncbi.nlm.nih.gov). The study was approved by the Ethics Committee of the Fondazione IRCCS Policlinico San Matteo (protocol no. 20080009620). Informed consent was obtained from all subjects prior to enrollment.

Viral RNA was extracted from serum samples using the automatic Easy Mag extractor (Biomerieux, Lyon, France), and full-length HCV NS3/4A sequences were amplified using a nested RT-PCR. In detail, the primers used respectively in PCR and nested PCR, spanning NS3/4A aa from 1 to 181, were as follows: 1a-Forward outer 5^′^-GACATCATCAACGGCTTGCCCG-3^′^ and 1a-Reverse outer 5^′^-GAGTACGTGATGGGGCTGCCAG-3^′^, 1a-Forward inner 5^′^-GGAATGGTCTCCAAGGGGTGGA-3^′^ and 1a-Reverse inner 5^′^-CATGGGCCTTGGACATGTAAGC-3^′^ for genotype 1a; 1b-Forward outer 5^′^-CGAGACCTTGCGGTGGCAGT-3^′^ and 1b-Reverse outer 5^′^-CAGCCGTYTCCGCTTGGTCC-3^′^, 1b-Forward inner 5^′^-CATCACCTGGGGGGCAGACACC-3^′^ and 1b-Reverse inner 5^′^-GTCAGTTGAGTGGCACTCATCAC-3^′^ for genotype 1b; 2abc-Forward outer 5^′^-GGCACHTAYATCTATGACCA-3^′^ and 2-Reverse outer 5^′^-CAGYCCRATGGAGARGAARGTCA-3^′^, 2-Forward inner 5^′^-GTYCTRATGTTGGGRTTGATBCC-3^′^ and 2-Reverse inner 5^′^-TASGCCCCAAAMCCMAGSGTGG-3^′^ for genotype 2; 3-Forward outer 5^′^-GTCTCTGCRCGATTAGGCCGTGA-3^′^ and 3-Reverse outer 5^′^-CAGTTTRGCACCAGTTGTAACG-3^′^, 3-Forward inner 5^′^-GTTGGGACCTGCTGATGACTA-3^′^ and 3-Reverse inner 5^′^-CCCAGTGCGGATGTTGGGGT-3^′^ for genotype 3; finally, 4-Forward outer 5^′^-GGGYAATGARATMYTGCTCGG-3^′^ and 4-Reverse outer 5^′^-GCCAGGAACTTMCCRTABGT-3^′^, 4-Forward inner 5^′^-GGAGRCTBCTYGCBCCCAT-3^′^ and 4-Reverse inner 5^′^-GAGTAYGTGATYGGCGC-3^′^ for genotype 4. The PCR products in the first round were obtained by using the following conditions: 15’ at 45°C for the reverse transcription followed by 10’ at 94°C, and then 50 cycles at 94°C for 1’, 55°C for 1’ and 72°C for 70”, with an extension at 72°C for 10’. Three microliters from the first PCR reaction were used in the nested PCR with the following conditions: denaturation step at 94°C for 10’ and then 30 cycles at 94°C for 1’, 52°C for 1’ and 72°C for 70”, with an extension at 72°C for 10’.

Direct sequencing of PCR products was performed using an automatic sequencer (ABI PRISM 3100 genetic analyzer DNA Sequencer, Applied Biosystems, Foster City, CA, USA) and the BigDye Terminator v1.1 Cycle Sequencing kit (Applied Biosystems, Foster City, CA, USA).

Only variants present in more than 5% of the patient virus populations for each HCV genotype group were considered in the genotypic resistance analysis [12].

Nucleotide sequences were assembled using the Sequencer 4.6 (Gene Codes Corp., Ann Arbor, MI) software program. To obtain a detailed subtyping of HCV strains, nucleotide sequences were aligned with confirmed references of different subtypes using the ClustalW method which is embedded in the Mega 5 package [20].

The phylogeny of the sequences was constructed using the Neighbour Joining method. The nucleotide substitution model was selected according to Akaike Information Criterion scores. A Neighbour Joining tree was constructed with MEGA 5 software [20] setting the Tamura 3-parameter as an evolutionary model with an heterogeneous rate among sites using gamma distribution for the relative rate. Branch support was assessed by bootstrap analysis with 1000 replicates. Bootstrap values of 70% were used as the cut off point for cluster analysis. The sequences reported in this study have been submitted to the GenBank database under accession numbers J × 170910 to J × 171065.

The GenBank accession numbers for reference sequences used to determine the HCV genotypes were as follows: M62321 (subtype 1a), NC004102 (subtype 1a; H77-US1977), D90208 (subtype 1b), D14853 (subtype 1c), D00944 (subtype 2a), D10988 (subtype 2b), D50409 (subtype 2c), AB031663 (subtype 2 k), D17763 (subtype 3a), D49374 (subtype 3b), D63821 (subtype 3 k), Y11604 (subtype 4a), GU085486 (subtype 4a), FJ025855 (subtype 4b), FJ025854 (subtype 4b), FJ462436 (subtype 4c) FJ462437 (subtype 4d), EU392172 (subtype 4d), EU392170 (subtype 4f), FJ462432 (subtype 4 g), FJ462438 (subtype 4 k), EU392171 (subtype 4 k) EU392173 (subtype 4 k), FJ839870 (subtype 4 l), FJ462433 (subtype 4 m), FJ462441 (subtype 4n), FJ462440 (subtype 4o), FJ462431 (subtype 4p), FJ462434 (subtype 4q) FJ462439 (subtype 4r) FJ839869 (subtype 4 t), Y13184 (subtype 5a), Y12083 (subtype 6a), D84262 (subtype 6b), D84263 (subtype 6d), D63822 (subtype 6 g), D84265 (subtype 6 h), D84264 (subtype 6 k).

## Results

The clinical and virologic characteristics of patients considered in the study are provided in Table 1. Fifteen patients (9.5%) were co-infected with HIV and treated with highly active antiretroviral therapy (HAART) (Table 1).

**Table 1:** Patient characteristics by HCV genotype

**Characteristic**	**HCV genotype**				
	**1a (n = 31)**	**1b (n = 39)**	**2 (n = 30)**	**3 (n = 33)**	**4 (n = 23)**
Gender					
Male	26 (83%)	20 (51%)	11 (36%)	26 (78%)	17 (85%)
Female	5 (27%)	19 (49%)	19 (64%)	7 (22%)	3 (15%)
Race					
Italian	29 (93%)	38 (97%)	30 (100%)	31 (93%)	13 (65%)
Others	2 (7%)	1 (3%)	0	2 (7%)	7 (35%)
No. of patients HIV-1 co-infected receiving HAART (%)	5	1	0	4	4
Median HCV viral load (IU/mL log_10_) in HCV mono-infected pts	5.66 (range 3.03-6.44)	5.63 (range 4.2-6.75)	5.96 (range 3.6-6.84)	5.38 (range 2.97-6.58)	5.66 (range 2.40-6.78)
Median HCV viral load (IU/mL log_10_) in HCV/HIV co-infected pts	6.41 (range 4.73-6.77)	5.95	0	6.54 (range 6.17-6.86)	6.45 (range 6.42-6.52)

Aa substitutions associated with HCV PI resistance were found in 50/156 (32%) sequences of PI naïve HCV patients (Table 2). Mutations V36L, T54S, V55A/I, and Q80K/L were observed in 29% of patients with genotype 1a, and V55F, Q80L/N and M175L in 10% of patients with genotype 1b. Mutation V158M was found in 3% patients with genotype 2, D168Q was present in 100% pts with genotype 3 and D168E was observed in 13% of patients with genotype 4 (Table 2). In addition, multiple aa polymorphisms not associated with PI resistance were detected in all genotypes (Table 2).

**Table 2:** Amino acid variations in the HCV NS3 protein associated with resistance mutations to HCV NS3 protease inhibitors, compensatory and enhanced replication

**NS3 Protease position**^**a**^	**HCV variation in different genotypes (number of sequenced patients)**^**b**^				
	**1a**	**1b**	**2**	**3**	**4**
	**(n = 31)**	**(n = 39)**	**(n = 30)**	**(n = 33)**	**(n = 23)**
36 (R)	**V36L**^**c**^**(2)**^e^	V36	L36	L36	L36
41 (R)	Q41	Q41	Q41	Q41	Q41
43 (R)	F43	F43	F43	F43	F43
54 (R)	**T54S (2)**^e^	T54	T54	T54	T54
55 (R)	**V55A/I (2)**^e^	**V55F (1)**^e^	V55	V55	V55
79 (R)	D79	D79	E79	D79	D79
80 (R)	**Q80K/L (3)**^e^	**Q80L/N (2)**^e^	G80	Q80	Q80
109 (R)	R109	R109	R109	R109	R109
138 (R)	S138	S138C (1)^d^	S138	S138	S138
155 (R)	R155	R155	R155	R155	R155
156 (R)	A156	A156	A156	A156	A156
158 (R)	V158	V158	**V158M (1)**^e^	V158	V158
168 (R)	D168	D168	D168	**D168Q (33)**^e^	**D168E (3)**^e^
170 (R)	I170	V170I (12)^d^	I170	I170V (1)^d^	V170
175 (R)	L175	**M175L (1)**^e^	L175	L175	L175
176 (R)	E176	E176	D176	S176N (4)^d^	E176
71 (C)	V71	**I71V/L (5)**^e^	V71	A71S (1)^d^	V71
72 (C)	**I72T/F (2)**^e^	**T72I/A/L (11)**^e^	T72	L72F (1)^d^	N72C (2)^d^
86 (C)	P86	**Q86P (6)**^e^	P86S (1)^d^	P86S (2)^d^	P86
88 (C)	P88	P88	P88	P88	P88

^a^R, position associated with primary resistance; C, position associated with compensatory mutations; (Lopez, 2008; Flint, 2009; Susser, 2009; Lentz, 2010; Verbimen, 2010; Romano, 2010, Halfon, 2011).

^b^Reference strains for each genotype: M62321 (1a), D90208 (1b), D50409 (2c), D17763 (3a), and Y11604 (4a).

^**c**^Letter on the left represents the wild type amino acid, on the right, the amino acid substitution. The number of patients with mutant HCV strains is indicated in brackets.

^d^Polymorphism with no associated resistance.

^e^ Amino acid changes conferring resistance are reported in bold.

Amino acids at positions S138 and V170 reported to be correlated with PI resistance when there is a change from T to T/A [7], respectively, changed to S138C and V170I in patients with genotype 1b were not associated with PI resistance. In addition, all sequences of genotypes 2, 3 and 4 showed the mutation V36L which has been associated with PI resistance in HCV patients with genotype 1. Of note, HCV PI resistance mutations in HCV/HIV co-infected patients were found in only one patient (Q80L).

A mutation at position 176 (S176N) different from that previously correlated with resistance (S176G) [11] was found in four patients with genotype 3. Previously reported [10] compensatory aa changes (I71V, I72T/F, and Q86P) were observed in both genotypes 1a and 1b. In individual genotype 1b strains, the resistance mutation V55F was associated with compensatory mutation T72I, mutation Q80L/N was associated with Q86P and mutation M175L was associated with T72I and Q86P. In contrast, the two compensatory mutations in genotype 1a were detected in the absence of resistance mutations. Finally, a number of polymorphisms not associated with PI resistance, were detected between codon 4 and codon 179 in all genotypes (Table 3). In genotypes 1a, 1b and 4 multiple polymorphisms (4, 15 and 29, respectively) were detected, while 11 and 17 polymorphisms were found in genotypes 2 and 3, respectively. In detail, the number of aa changes for each natural polymorphic site in the different genotypes was 104 in genotype 1a, 186 in genotype 1b, 84 in genotype 2, 97 in genotype 3 and 255 in genotype 4.

**Table 3:** Amino acid variations in the HCV NS3 protein not associated with resistance mutations to HCV NS3 protease

**NS3 Protease position**^**a**^	**HCV variation in different genotypes (number of sequenced patients)**^**b**^					**NS3 Protease position**^**a**^	**HCV variation in different genotypes (number of sequenced patients)**^**b**^				
	**1a (n = 31)**	**1b (n = 39)**	**2 (n = 30)**	**3 (n = 33)**	**4 (n = 23)**		**1a (n = 31)**	**1b (n = 39)**	**2 (n = 30)**	**3 (n = 33)**	**4 (n = 23)**
**4**					T4P (14)	**67**	S67P/A (14)			A67V/T (7)	
**5**				A5T/P (3)	A5G/C(6)	**69**				H69R (2)	
**7**		S7A (12)	A7T/V (11)	A7T(2)		**72**	I72T/F (2)	T72A/I/L (13)			
**10**					T10H/N (2)	**82**		L82G (3)			
**11**					R11P (2)	**83**		V83N/T/I			
**12**					G12A/R (3)	**91**	S91A/T (28)	A91S (5)		A91T (4)	
**13**					L13M/W/T (13)	**92**		T95E/S (2)			R92K/T (15)
**14**		L14F (5)		L14F (3)	F14L/I (15)	**95**				E95D (3)	A95T/S (4)
**15**			D15G (28)		S15G (12)	**98**				A/98 T (15)	
**16**			A16T (8)			**101**					S101A (8)
**18**	I18V (2)	I18V (4)			V18I (3)	**102**				S102A (3)	A102S (20)
**20**				S20G (4)		**105**					Y105F (14)
**24**				R24K (2)	R24K (3)	**107**		V107I (4)			
**26**		K26R (3)				**110**				D110E (5)	H110N (3)
**28**	Q28E/L (3)		D28E (2)	V28I/M (5)		**114**	I114V (3)	V114I (26)			I114V (18)
**33**	V33I (2)		V33I (10)		V33I (13)	**117**		R117H/C (4)			
**35**		V35I (5)	V35I (3)			**119**			R119Q (2)		
**39**		A39T (3)				**122**	S122G (10)	S122T/C (5)			T122S (2)
**40**	A40T/S (9)					**123**		R123K (3)			
**42**		S42T/F (5)				**127**					L127I (11)
**44**					L44M (2)	**132**		I132V/M (18)		L132I (2)	
**46**		T46S/A (2)				**134**			S134T (13)		
**47**					A47G/S (2)	**146**			P146S (2)		
**48**	I48V (4)	V48I/L (14)			V48I (11)	**147**		S147L (2)		S147L/A/R/T (5)	M147L/Q (15)
**49**		N49S (2)				**150**		V150A (29)			R150V/A (20)
**51**		V51A (2)	V51T/A (3)			**151**					A151V (3)
**56**		Y56F/C (4)				**153**		I153V/L (3)			
**60**		S60A/T/P (7)				**166**				S166A/T (3)	
**61**	T61S (2)	K61R (4)				**174**	N174S/G (23)	S174A/L (3)	S174T (2)		
**64**	I64L (2)					**177**			V177I (3)		
**65**					S65C (2)	**179**				A179T/V (2)	

^a^Amino acid position associated with polymorphisms compared to reference sequences;

^b^Reference strain accession numbers for each genotype: M62321 (1a), D90208 (1b), D50409 (2c), D17763 (3a), and Y11604 (4a).

^**c**^Letter on the left represents the wild type amino acid, on the right, the amino acid substitution. The number of patients with mutant HCV strains is indicated in brackets.

**Figure 1:**
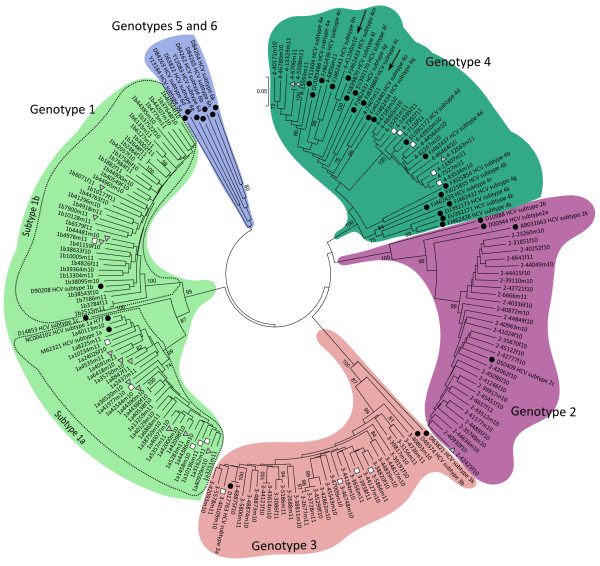
**Phylogenetic analysis of HCV protease in PI-naïve patients.** Black circles ● indicate available reference sequences; grey triangles ▲, protease baseline resistant patients; white squares □, HIV co-infected patients.

The mean genetic diversity of NS3 was higher in genotype 4 (16.6%) than in genotype 1b (12.0%), 1a (10.4%), 2 (11.2%), and 3 (9.2%). Among patients infected with HCV genotype 1, sequences were equally distributed in HCV subtypes 1a and 1b. The number of sequences carrying mutations associated with PI resistance was 2-fold higher in subtype 1a with respect to subtype 1b (p = 0.07) (Figure 1). All sequences clustering within genotype 2, belonged to subtype 2c and only one sequence carried a mutation correlated with resistance. All sequences from patients infected with genotype 3 clustered in the subtype 3a, and all sequences showed the D168Q change (Figure 1). Among the HCV genotype 4 sequences, 14/23 (60.9%) belonged to subtype 4d, while, 6/23 (26.1%) were subtype 4a, 1/23 (4.3%) was subtype 4c and 2/23 (8.7%) clustered together with an uncommon subtype (Figure 1). Among these, one sequence exhibited 91.6% identity with HCV subtype 4 m, and the second exhibited 89.1% identity with HCV subtype 4 t (bootstrap value >99%) (Figure 1).

All HCV/HIV co-infected patients with genotype 4 clustered in the subtype 4c, while the mutations correlated with PI resistance were observed both in subtype 4c and subtype 4a (Figure 1). In particular, a mutation associated with PI resistance (D168E) was observed in two identical sequences (4-6706 m11 and 4-5208 m11) from different patients.

## Discussion

The identification of baseline resistance mutations to anti-HCV PIs is crucial for defining new therapeutic approaches. Natural polymorphisms in the HCV NS3/4A protease-coding region were analyzed in 156 patients including genotypes 1a, 1b, 2, 3, and 4. Relevant natural aa polymorphisms were found among the different genotypes and subtypes. The data presented are important not only to determine whether PI-resistant mutants are likely to be present in PI treatment-naïve patients, but also for the examination of HCV protease among different genotypes and the possibility of eventually extending the PI treatment to non-genotype 1-infected patients. On the other hand, the study of preexisting viral variants to predict response to PIs for genotypes other than 1 might be misleading, since the molecular target structure could be considerably different between genotype 1 HCV and other genotypes. In fact, all recent clinical trials have been designed for treatment of HCV infections with genotype 1 [5,6,21]. Although culture systems for determining HCV susceptibility to PI compounds have been recently developed [4], the comparative genetic analysis of HCV strains in PI naïve patients infected with different virus genotypes may provide information useful for predicting treatment efficacy since, naturally occurring genotype-specific variations appear to have an effect in different HCV genotypes [22,23]. In addition, even though resistant viral variants exist at low frequency in untreated patients, specific NS3 protease mutations may have an important role in modulating resistance development and modifying viral fitness [22].

Substitutions at positions R155 and A156, which are known to confer a high level of resistance to all PIs [7,9], were not observed in our patients. In contrast, other minor mutations conferring low levels of resistance to PIs [7,9,14,22] were found in 32% of patients. In addition, all sequences from genotypes 2, 3 and 4 showed the V36L mutation which is known to confer decreased susceptibility to telaprevir [7,24] and all sequences from genotype 3 showed the D168Q mutation which is known to decrease the activity of non-covalent HCV NS3 protease inhibitors against genotype 3 (4, 14). Moreover, a higher number of polymorphic sites in HCV protease NS3/4A were observed in genotypes 1b and 4 compared with genotypes 1a, 2 and 3. Further studies are needed to better understand the potential implications on treatment of PI naïve patients with resistance at baseline which could influence the treatment failure rate. On the other hand the clinical role of compensatory mutations impacting the viral fitness of PI resistant strains [25-27] requires additional investigation.

In contrast with reported observations [28], HCV PI resistance mutations were not observed more frequently in HCV/HIV co-infected patients than HCV mono-infected patients. Phylogenetic analysis confirmed the greater heterogeneity of HCV genotypes 1b and 4, which may be explained by the presence of several divergent sequences with respect to other genotypes. In keeping with data from the Italian HCV genotype distribution [29], subtype 4d strains were observed also in our series. In this data set, a wide distribution of mutations correlating with PI resistance was observed in all genotypes. A larger data set of HCV sequences including baseline data would clarify this finding.

In conclusion, i) the natural variability in all HCV viral populations (HIV co-infected or mono-infected) observed in our study confirms [30-32] and underlines their potential implication in the management of HCV treatment; ii) no major mutations associated with resistance to PIs were observed in HCV PI naïve patients, on the contrary, a consistent number of minor mutations which may reduce the efficacy of PIs were detected in genotypes 1a, 1b and also in genotypes 2, 3 and 4. Thus, the inclusion of patients with different genotypes in future larger clinical trials, would help define the efficacy of anti HCV PIs in patients infected with different genotypes and these data could be extended to design treatment protocols. In addition, further investigations are necessary to understand the utility of resistance analysis at baseline to evaluate response to HCV protease inhibitors.

## Consent

Written informed consent was obtained from patients for publication of this manuscript and any accompanying images. A copy of the letter of consent is available for review by the Editor-in-Chief of this journal.

## Competing interests

The authors declare that they have no financial or competing interests.

## Authors’ contributions

SP study design, data analysis and paper writing. LF, AP, MG, LD sequencing and phylogenetic analysis. RG, SN, GB, PS patient enrolment. FB data analysis, manuscript revision and fund raising. All authors read and approved the final manuscript.
